# Whole-Genome Sequence and Annotation of Octopine-Utilizing *Pseudomonas kilonensis* (Previously *P. fluorescens*) Strain 1855-344

**DOI:** 10.1128/genomeA.00463-15

**Published:** 2015-05-14

**Authors:** Wilhelm Wei Han Eng, Han Ming Gan, Huan You Gan, André O. Hudson, Michael A. Savka

**Affiliations:** a Monash University Malaysia Genomics Facility, Monash University Malaysia, Bandar Sunway, Petaling Jaya, Selangor, Malaysia; b School of Science, Monash University Malaysia, Bandar Sunway, Petaling Jaya, Selangor, Malaysia; c Thomas H. Gosnell School of Life Sciences, Rochester Institute of Technology, Rochester, New York, USA

## Abstract

Here, we report the whole-genome sequence and annotation of *Pseudomonas kilonensis* 1855-344 (previously known as *P. fluorescens* 1855-344). The genome contains an octopine oxidase gene cluster consistent with the ability to utilize octopine. A biosynthetic gene cluster was identified for mangotoxin and aryl-polyene using the antiSMASH server.

## GENOME ANNOUNCEMENT

The catabolism of unique carbon compounds collectively called opines produced in plant neoplasias as a result of transformation by *Agrobacterium tumefaciens*, *Allorhizobium vitis* (previously *A. vitis*), and *Rhizobium rhizogenes* (previously *A. rhizogenes*) is rare in microorganisms outside of the *Rhizobiaceae* (1). Here, we report the whole-genome sequence and annotation of the *Pseudomonas fluorescens* strain 1855-344. Strain 1855-344 exhibits an unusual metabolic trait of utilizing the opine, octopine (2). Strain 1855-344 has been used to study the opine concept in *Agrobacterium*-plant interactions (1, 3) and for genetic and bio- chemical studies on opine catabolic traits (4) and as a host for a novel genetic tag for environmental tracking (5). This work presents the genome of the first opine-utilizing microorganism that is not a member of the *Rhizobiaceae*, a genome-based taxonomic revision, and annotation analyses that uncover the synthesis of bioactive compounds in addition to the identification of the opine catabolic gene cluster.

Genomic DNA was isolated using the Omega Biotek EZDNA tissue DNA kit (Omega Biotek, Norcross, GA). The purified DNA was prepared with Nextera XT (Illumina, San Diego, CA) and sequenced on the MiSeq (2 × 250 bp) at the Monash University Malaysia Genomics Facility. The FASTQ files were adapter-trimmed with Trimmomatic 0.33 and *de novo* assembled with SPAdes 3.5.0 (6–8). The generated contigs were scaffolded and gap-filled using SSPACE 3.0 and GapFiller 1.10, respectively (9, 10). Average nucleotide identify was calculated using JSpecies (11). The final assembly consists of 73 contigs with a total length of 6,843,717 bp (G+C content, 60.7% and *N*_50_ of 166,355 bp) and an average coverage of 117×. Annotation was done with NCBI Prokaryotic Genome Annotation Pipeline, leading to the prediction of 5,856 open reading frames, 8 rRNAs, and 61 tRNAs.

The 16S rRNA and *rpoB* gene sequence of strain 1855-344 is 99.87% and 98.74% identical to that of *Pseudomonas kilonensis* type strain 520-20 (Genbank accession no. AJ292426 and AJ717472, respectively). Further, strain 1855-344 did not display >95% average nucleotide identity with *P. brassicacearum*, a very close relative of *P. kilonensis* with borderline DDH value thus supporting to the designation of *P. kilonensis* as a valid species (12). The octopine-catabolic operon, *ooxAB*, of strain 1855-344 is located in contig17 as suggested by high blast scores of predicted proteins against various Swiss-Prot-reviewed OoxA and OoxB proteins and the presence of signature protein domains such as PIRSF037495 and PF01266 (2, 13–16).

Using the antibiotics and secondary metabolite analysis shell (antiSMASH) (17, 18), a total of 14 gene clusters for putative biosynthetic secondary metabolites were predicted. These are distributed into genes for five nonribosomal peptide synthetases (NRPSs) and three gene clusters involved in bacteriocin biosynthesis. AntiSMASH also predicted clusters for linaridin, butyrolactone, lantipeptide, and aryl-polyene. The clusters with the highest percentage of genes that show homology were mangotoxin with 57% of the genes located in contig40 between nucleotides 28,916 and 65,712 and APE Vf aryl-polyene with 40% of genes located in contig29 between nucleotides 34,480 and 78,091.

### Nucleotide sequence accession numbers. 

The nucleotide sequences have been deposited at DDBJ/EMBL/GenBank under accession no. JZXC00000000. The BioProject number is PRJNA277995 and the BioSample number is SAMN03401053.
